# Improving the Recognition Accuracy of Memristive Neural Networks via Homogenized Analog Type Conductance Quantization

**DOI:** 10.3390/mi11040427

**Published:** 2020-04-18

**Authors:** Qilai Chen, Tingting Han, Minghua Tang, Zhang Zhang, Xuejun Zheng, Gang Liu

**Affiliations:** 1 School of Mechanical Engineering, Xiangtan University, Xiangtan 411105, China; chenqilai8816@126.com; 2 School of Electronic Information and Electrical Engineering, Shanghai Jiao Tong University, Shanghai 200240, China; hantt1234@126.com; 3 School of Electronic Science and Applied Physics, Hefei University of Technology, Hefei 230601, China; zhangzhang@hfut.edu.cn; 4 School of Materials Science and Engineering, Xiangtan University, Xiangtan 411105, China; tangminghua@xtu.edu.cn

**Keywords:** memristors, quantum conductance, neural networks, pattern recognition

## Abstract

Conductance quantization (QC) phenomena occurring in metal oxide based memristors demonstrate great potential for high-density data storage through multilevel switching, and analog synaptic weight update for effective training of the artificial neural networks. Continuous, linear and symmetrical modulation of the device conductance is a critical issue in QC behavior of memristors. In this contribution, we employ the scanning probe microscope (SPM) assisted electrode engineering strategy to control the ion migration process to construct single conductive filaments in Pt/HfO_x_/Pt devices. Upon deliberate tuning and evolution of the filament, 32 half integer quantized conductance states in the 16 G_0_ to 0.5 G_0_ range with enhanced distribution uniformity was achieved. Simulation results revealed that the numbers of the available QC states and fluctuation of the conductance at each state play an important role in determining the overall performance of the neural networks. The 32-state QC behavior of the hafnium oxide device shows improved recognition accuracy approaching 90% for handwritten digits, based on analog type operation of the multilayer perception (MLP) neural network.

## 1. Introduction

Resembling the operating principle of human brains that transmit and process information through huge amounts of interconnected neurons and synapses [1,2,3,4], neuromorphic computing paradigm based on new solid-state electronic devices have demonstrated advantages of high efficiency, low power consumption, and parallel processing ability when handling big-data analysis tasks [5,6,7]. Plenty of research efforts have been devoted to emulating the electrical functions of biological synapses and neurons with ferroelectric, magnetic, phase-change, and resistive switching devices [8,9,10], wherein the resistive switching memristors distinguish themselves as promising post-Moore era candidates with their simple device structure, crossbar array, and 3D stacking capability for very large scale integration [11,12,13,14,15,16,17]. In particular, the ion migration and filamentary conduction mechanism make the memristor devices extremely scalable, enabling them to easily approach the lithographic limitations [18,19,20,21]. It was demonstrated that the computing and energy efficiency of memristor-based in-memory computation was comparable to that of the complementary metal-oxide-semiconductor (CMOS) platforms [22,23].

Shrinking the dimension of the conductive filaments (CFs) into the atomic scale of quantum point contact allows memristor ballistic electron transport without scattering and quantized conductance (QC) characteristics in analog domains [24,25,26]. It not only significantly increases the data storage capacity of the devices, but also allows stronger information processing ability in neuromorphic systems. Generally, multiple conductance states of the memristor devices should be achieved in ways that are as simple as possible for practical usage [27,28,29,30]. Nonetheless, most of the studies until now were implemented with complex programming schemes to achieve multi-conductance characteristics, in which the involvement of varying current compliance and voltages leads to a heavy burden on the system circuit design [31,32,33,34]. Advancements are highly desired to simplify the operating philosophy and realize reliable and analog type conductance quantization behavior in resistive switching memristors.

In this contribution, we report an effective approach to regulate the multilevel quantized conductance characteristics through pre- and customized formation of the conductive filament in a controllable manner in metal oxide based memristor devices. By inducing the directional migration of oxygen anions under the stress of a concentrated electric field through a conductive scanning probe microscopic (SPM) tip, indentation and consequently protrusion of the metal electrode into the switching matrix can cause the construction of a single conductive filament at a fixed position with better controllability. Thirty-two continuous quantized conductance states can be obtained by deliberately manipulating the as-formed CF, which gives rise to ≈20% enhancement in the uniformity of conductance value distribution in each QC state. More importantly, enhanced recognition accuracy approaching 90% can be achieved by multi-layer perception, employing HfO_x_ memristor with homogenized, analog type conductance quantization for both handwritten digit patterns.

## 2. Materials and Methods

**Figure 1:**
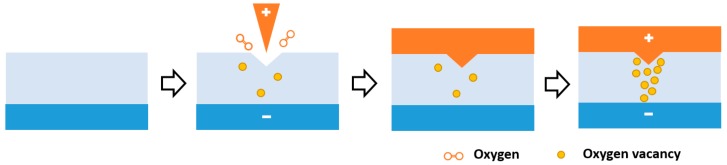
Flow chart of device fabrication assisted by scanning probe microscope technique.

The Pt/HfO_x_/Pt sandwich structured memristor devices were fabricated on commercial Pt/Ti/SiO_2_ wafers (HF-Keijing, heifei, China) by depositing 10 nm HfO_x_ thin film through RF magnetron sputtering technique in a pure argon environment, with an environmental pressure of 1 Pa and using high purity HfO_2_ ceramic (99.995%) as the target. For samples with a flat Pt/HfO_x_ interface, the top platinum electrodes, with a thickness of 50 nm, were directly deposited onto the HfO_x_ nanofilm by electron beam evaporation at a pressure of ≈10^−6^ Pa and a deposition rate of ≈0.5 Å/s at room temperature. For samples with inward conical Pt electrode protrusion, a layer of photoresist (AZ-5214E, QiYao Opto-Electronics, ShenZhen, China) was first spin-coated onto the HfO_x_ nanofilm, followed by photolithography patterning using a positive photomask and lift-off process to define an exposed HfO_x_ area with a radii of 100 μm. Then a Pt-coated scanning probe microscope tip was placed onto the surface of the circular HfO_x_ exposed area in contact mode, where local voltage sweeps with different amplitudes and loading cycles were applied onto the sample to drive the migration of oxygen anions/vacancies to form indentations (Figure 1). During operation, the bottom Pt electrode was always grounded, and all the operations were conducted in ambient environment. Afterward, Pt top electrodes were deposited into the circular pattern through E-beam evaporation.

## 3. Results and Discussion

**Figure 2:**
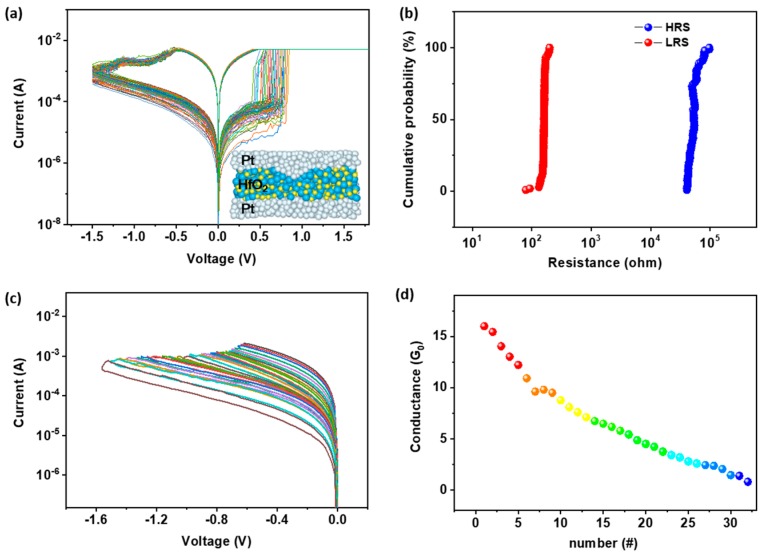
(**a**) Current–voltage characteristics of Pt/HfO_x_/Pt memristor device showing resistive switching with an ON/OFF ratio exceeding 10^3^. Inset shows the structure of the device, with electrode protrusion extending into the hafnium oxide switching layer. (**b**) Histogram of the device resistances in the ON and OFF state. (**c**) Continuous regulation of the device current in the negatively biased reset processes. (**d**) Evolution of the device conductance as a function of the voltage pulse stressing numbers.

Variation of the switching parameters, including the programming voltages and device resistances in different QC states, is the main cause of performance deterioration in terms of operating reliability and recognition accuracy of the memristor based neural networks. It can be ascribed to the random ion migration in the polycrystalline metal oxide switching matrix, and consequently the stochastic nature of the branch-shaped multiple conductive filament formation, disruption, and regeneration during cyclic operations [35,36]. In order to achieve a more stable and adjustable conductance state for memristor devices, we performed pre-treatment of the HfO_x_ switching layer using the scanning probe microscope based electrochemical lithography technique [37,38]. By stressing voltages onto the hafnium oxide layer through a conductive SPM tip, the highly localized electric field formed under the tip pinpoint region can induce directional migration of the mobile oxygen vacancies towards the tip position, resulting in loss of the local mass, and therefore formation of a concave structured indentation. As demonstrated in our previous study [38], capping the top Pt electrode formed a downward pointing metal protrusion into the switching layer, which acted as a microelectrode that concentrated the internal electric field distribution and led to the formation of a single conductive filament in the memristor device. The evolution of the as-formed CF is more controllable, so the reliability of the device can be greatly improved. As shown in Figure 2a, scanning the pre-treated Pt/HfO_x_/Pt device between −1.5 V and +2 V with a current compliance preset of 10 mA can produce resistive switching characteristics, showing promising cycling uniformity. For over hundreds of switching cycles, both the ON and OFF state resistances were distributed in a narrow range (Figure 2b). A high ON/OFF ratio exceeding 10^3^ can be maintained reliably, allowing a wide regulation window for the achievement of conductance quantization, with sufficient resolution for differentiating the adjacent QC states for multilevel operations. Theoretically, the ON and OFF device resistances of 35,000 Ω and 300 Ω corresponds to the conductance modulation range of 0.5 G_0_–50 G_0_, which is even broader than that used in synaptic weight updating of the reported memristive neural networks [39,40]. Herein, G_0_ is the integer unit of quantized conductance, 77.5 μS. Similar pre-treatment can be performed with the nano-imprint lithography (NIL) technique during large scale fabrications.

Conductance quantization can be realized by either controlling the current compliance in the set processes or changing the cut-off voltages of the reset processed [41,42]. Since the positive feedback of the set procedure usually led to uncontrolled overgrowth of the conductive filament with overshooting device conductance, or even the absence of the QC states [37], we employed a relatively more moderate reset process to modulate the evolution of the conductive filament. As depicted in Figure 2c, resetting the Pt/HfO_x_/Pt device with increasing stopping voltages of −0.6 V to −1.6 V during direct current (DC) scanning can consecutively decrease the device currents. Replotting in the conductance vs. number of scanning curve, or the conductance vs. number of pulse stressing curve, reveals a continuous modulation of device conductance from 16 G_0_ to 0.5 G_0_ in a half-integer QC step (Figure 2d). The pulse-mode measurement was conducted by applying voltage pulses with the width of 10 ms and increasing amplitudes from −0.6 V to −1.6 V. For both the DC scanning and pulse stressing operations, the ramping steps of the voltages are −0.02 V in the −0.6 V to −0.84 V range, with an increase to −0.04 V in the −0.84 V to −1.6 V range. This, nevertheless, is consistent with the negative feedback characteristics of the reset process, wherein the shrinking of the conductive filament dimension with smaller device currents will slow the Joule heating related modulating process. In total, 32 quantized conductance states were obtained in a stepwise manner in the DC scanning or pulse stressing mode. When the conductance goes beyond this range, the conductive filament becomes so thick that significant Joule heating with large device currents can annihilate the CF easily, resulting in a mutant reset process with partial absence of the QC states with larger conductance values. On the other hand, as the device conductance decreases to less than 1 G_0_, the atomic point contact gets completely disconnected to the metal electrodes, and the quantum conductance effect does not exist any longer. Nevertheless, it is noteworthy that the one directional update of the synaptic weight in the reset process may lead to additional complexity in the operating methodology or circuit design during practical applications. For instance, potentiation of the synaptic weight can be only achieved by a combination operation of binary switching of the device to a high conductance state, and subsequent depression to the desired level in the negatively biased reset process [38,39]. Therefore, symmetrical conductance modulation and fast blind weight updating are more desired in warranting the computing efficiency of the neural network.

**Figure 3:**
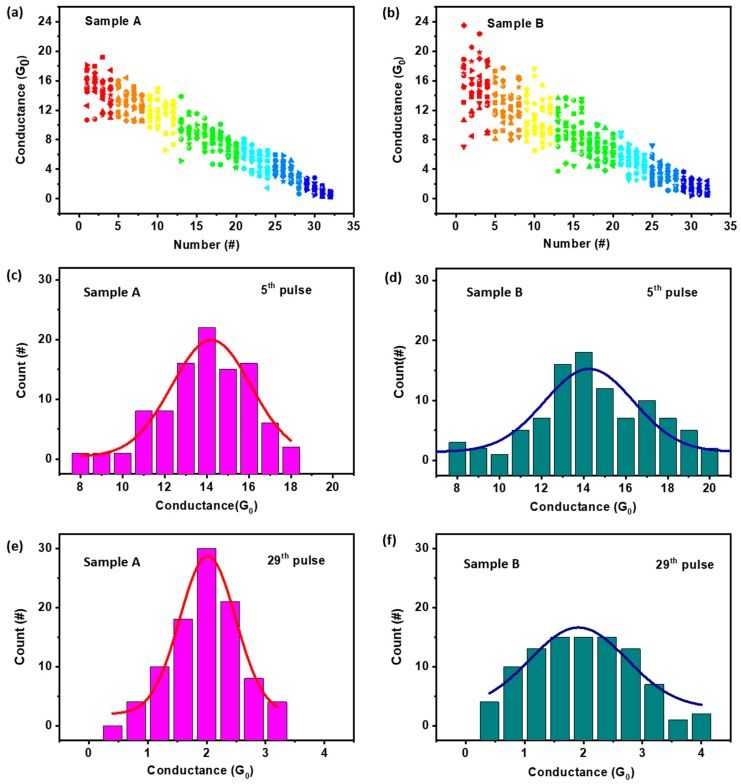
Evolution of the device conductance as a function of the pulse modulating numbers for (**a**) the pre-treated Pt/HfO_x_/Pt memristor device sample A with microelectrode protrusion and (**b**) the untreated control device sample B with flat Pt/HfO_x_ interface. Histogram and Gaussian curve fitting of the device conductance distributions at the 5th and 29th pulse modulation stages for sample A (**c**,**e**) and sample B (**d**,**f**), respectively.

Although the present electrode engineering strategy does not guarantee that each step of the modulation will strictly undergo a 0.5 G_0_ change during the cyclic operations, linear evolution of the device conductance benefiting the synaptic weight update for training the neural network can still be received, as shown in Figure 3a. The device conductance was modulated from 16 G_0_ to 0.5 G_0_ continuously in pulse-mode operation and set by the positively biased voltage scanning from 0 V to 1 V with a current compliance of 5 mA to reprogram the Pt/HfO_x_/Pt memristor to ON state. Afterward, the negative voltage pulse modulation was reconducted for a total of 12 times to give the data plotted in Figure 3a. All of the 32 QC states can be obtained repeatedly, however, the nominal conductance values varied in a small range (e.g., 33.26% for 16 G_0_) during cyclic modulation. For control sample B, without pre-treatment of the HfO_x_ layer or top electrode protrusion, a linear but more discrete distribution of the device conductance was observed at each stage of the pulse-mode modulations (Figure 3b). The variation of sample B’s nominal conductance approached 55.95% for 16 G_0_. Therefore, it was more likely to reach the desired quantized conductance within the pre-treated devices, wherein the formation of protruding microelectrodes can effectively regulate the evolution of a single conductive filament (rather than multiple CFs) in a more homogenized manner. Increasing the sampling number of both kind of devices can reveal this feature more obviously. For instance, at the fifth pulse modulation state, the FWHM (full wave at half maximum) of the Gaussian curve fitting of device A’s conductance distribution around the nominal value, 14 G_0_, was 4.4 (Figure 3c); whereas the respective number increased to 5.5 for sample B (Figure 3d), suggesting that a 25% enhancement in the uniformity of the conductance value can be made possible by the present SPM pre-treatment method. Similarly, the statistics collected at the 29th pulse modulation state (for conductance of 2 G_0_) shows FWHMs of 1.2 for sample A, and a much larger value of 2.3 for sample B.

**Figure 4:**
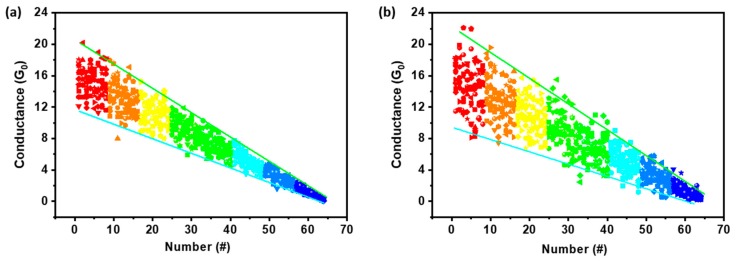
Mathematically simulated distribution of the device conductance in the 16 G_0_ to 0.5 G_0_ range with a 0.25 G_0_ step for (**a**) the pre-treated Pt/HfO_x_/Pt memristor device sample A with microelectrode protrusion and (**b**) the untreated control device sample B with flat Pt/HfO_x_ interface, respectively.

Further analysis of the collected data for both samples A and B with the normal distribution function (NDF) allows us to receive an equation,

(1) $$G=\left(1-kn\right)\times f\left(\mu ,\sigma^{2}\right),$$

to mathematically predict the device conductance at certain pulse modulation stages, where *G* is the varying device conductance, *n* represents the number of the pulses applied, *k*, *σ*, and *μ* reflect the variation range, variability, and standard value of the device conductance at each modulation state, and *f*(*μ*, *σ^2^*) is a general number generator that obeys the NDF’s law. Upon setting *k* = 0.03, *σ* = 1, and *μ* = 16.5 − 0.5*n* for sample A, and *k* = 0.03, *σ* = 4, and *μ* = 16.5 − 0.5*n* for sample B, faithful reproduction of the device conductance for 12 continuous pulse modulation cycles can be obtained through the mathematical simulation shown in Figure 4. All the simulated datasets fall in the experimental range depicted in Figure 3a,b, suggesting that modeling with the above equation and parameters can well resemble evolution processes of the Pt/HfO_x_/Pt memristor devices, with either microelectrode protrusion or flattening of electrode/oxide interfaces. As such, considering the deviation of the atomic point contact’s composition and geometry from pure metallic hafnium-based cones, which is probably the case during device operation by atomic exchange with the surrounding HfO_x_ matrix under the concentration gradient, fractional conductance quantization can also be modelled accordingly. This may offer additional guidance for deliberate tuning of the device electrical performance to receive more (e.g., 64) levels of linearly and symmetrically modulated conductance, which can lead to analog type operation and enhanced accuracy for pattern recognition with the memristive neural networks.

**Figure 5:**
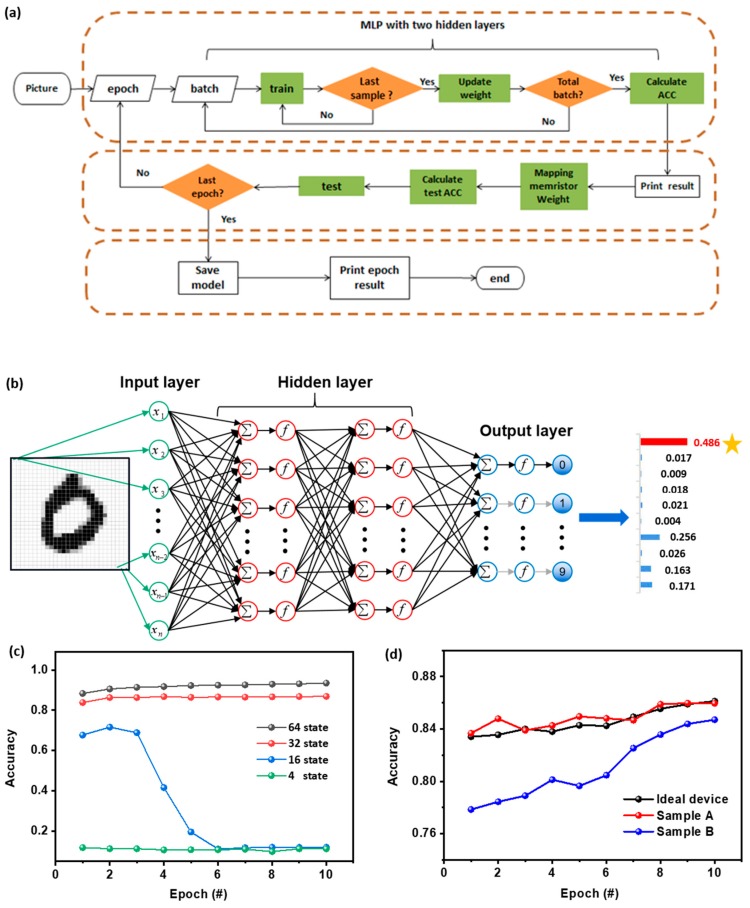
(**a**) Schematic flowchart for the simulation of supervised learning with multilayer perception (MLP) neural network for handwritten digit recognition. (**b**) Architecture of the MLP network. Plots of the recognition accuracy as a function of the increasing training epochs (**c**) with the available QC states numbers of 4, 16, 32 and 64 in sample A and (**d**) with 32 QC states in sample A, B, and ideal devices without conductance fluctuation.

For demonstration, simulation of supervised learning consisted of the offline training of the multilayer perception (MLP) neural network, synaptic weight updating in the memristor array, and tests of the handwritten digit pattern recognition, and was carried out using the experimental quantized conductance characteristics with back propagation (BP) algorithm (Figure 5a). Seven hundred and eighty four 28 × 28 pixel images of handwritten digits are obtained from the Keras database and utilized for training and recognition tests [43]. Accordingly, the as-constructed MLP network contained 784 input neurons, two-layer arrangement of 16 × 16 (256) hidden neurons, and 10 output neurons, with respect to the digits of 0 to 9 (Figure 5b). During the training courses, quantized conductance characteristics with 4, 16, 32, and 64 states were employed to renew the synaptic weights. Actual conductance values were considered and transferred to the synapse array upon normalization. Figure 5c displays the variation of recognition accuracy, along with the increased training epochs and numbers, of the available QC states. As shown, the less effective weight updating of the MLP network with four levels of QC characteristics can only lead to ≈10% recognition of the handwritten digits, while increasing the numbers of the available QC states to 16 may improve the accuracy to 71.2%. Nevertheless, it was observed that as the training courses continue, the recognition rate suddenly drops to ≈10% after six epochs. This can be ascribed to the fact that small numbers of the available QC states may result in lesser amounts of conductance levels available for weight updating and thus larger learning gradients and undesired convergence of the training loss function at the local minimum, which in turn makes the neural network unable to perform the multiplication-and-accumulation (MAC) operations any longer. As a consequence, the training process fails. When the numbers of the QC states reach 32 and 64, the analog manner of the MLP network operation shows promising pattern recognition accuracy of 86.8% and 93.5% after 10 training epochs, respectively, again suggesting the numbers of conductance states will directly affect the final recognition accuracy of the neural network. Only when the conductance levels are higher than 32, the accuracy of the QC based MLP network can be steadily improved with the number of trainings. It was also noteworthy that in the present study the employment of 1-bit operation technically limits the overall computing accuracy of the network. In the case that multi-bit inputs can be projected onto multiple memristor cells, greatly enhanced accuracy can be possible [44,45,46].

To further confirm the influence of electrode engineering on the recognition accuracy of the MLP network, we use the 32-state QC characteristics of samples A and B for the supervised learning simulation. The normal distribution formula was adopted to simulate the conductance value fluctuation during the synaptic weight updating procedure. As shown in Figure 5d, the black curve plotted with idealized conductance values at each QC state (*σ* = 0) displays a recognition accuracy of ≈86.1% for handwritten digits. Minor deviation from the ideality does not affect the performance of the network significantly, and the red curve simulated with the memristive characteristics of sample A gives a comparable recognition rate of ≈86.0%. For the case of sample B without electrode engineering, although the accuracy rises rapidly as the training course continues (blue curve), there was still an obvious performance gap of ≈2% after 10 epochs, when in comparison with the network constructed from the ideal or sample A memristor devices. Therefore, the fluctuation occurring during device conductance modulation, beyond the promising linearity in synaptic weight updating, plays an important role in improving the overall performance of the memristive neural networks. Reduced fluctuation of the switching parameters can project the trained network onto the memristor array with higher learning accuracy, which in turn guarantees the fidelity of MAC operation and BP algorithm more reliably.

## 4. Conclusion

In this work, we demonstrate a reliable hafnium oxide based memristor device that displays homogenized conductance quantization characteristics with 32 half-integer QC states. Through deliberate design of the electrode/switching matrix interface with the assistance of a SPM tip, a single conductive filament can be generated inside the memristive layer, which suppresses the evolution randomness of the multiple CFs and enhances the uniformity of the device conductance effectively. Simulation results indicate that with the linearly and symmetrically modulated QC behavior with conductance states of 32 levels, improved pattern recognition accuracy approaching 90% can be achieved through analog type operation of the multilayer perception neural network with the present memristor devices.
